# Flavonoids kaempferol and quercetin are nuclear receptor 4A1 (NR4A1, Nur77) ligands and inhibit rhabdomyosarcoma cell and tumor growth

**DOI:** 10.1186/s13046-021-02199-9

**Published:** 2021-12-14

**Authors:** Rupesh Shrestha, Kumaravel Mohankumar, Greg Martin, Amanuel Hailemariam, Syng-ook Lee, Un-ho Jin, Robert Burghardt, Stephen Safe

**Affiliations:** 1 Department of Biochemistry and Biophysics, Texas A&M University, College Station, TX 77843 USA; 2 Department of Veterinary Physiology and Pharmacology, Texas A&M University, 4466 TAMU, College Station, TX 77843-4466 USA; 3 Department of Food Science and Technology, Keimyung University, Daegu, 42601 Republic of Korea; 4 Department of Veterinary Integrated Biosciences, Texas A&M University, College Station, TX 77843 USA

**Keywords:** Kaempferol, Quercetin, Rhabdomyosarcoma, NR4A1, Anticancer

## Abstract

**Background:**

Flavonoids exhibit both chemopreventive and chemotherapeutic activity for multiple tumor types, however, their mechanisms of action are not well defined. Based on some of their functional and gene modifying activities as anticancer agents, we hypothesized that kaempferol and quercetin were nuclear receptor 4A1 (NR4A1, Nur77) ligands and confirmed that both compounds directly bound NR4A1 with K_D_ values of 3.1 and 0.93 μM, respectively.

**Methods:**

The activities of kaempferol and quercetin were determined in direct binding to NR4A1 protein and in NR4A1-dependent transactivation assays in Rh30 and Rh41 rhabdomyosarcoma (RMS) cells. Flavonoid-dependent effects as inhibitors of cell growth, survival and invasion were determined in XTT and Boyden chamber assays respectively and changes in protein levels were determined by western blots. Tumor growth inhibition studies were carried out in athymic nude mice bearing Rh30 cells as xenografts.

**Results:**

Kaempferol and quercetin bind NR4A1 protein and inhibit NR4A1-dependent transactivation in RMS cells. NR4A1 also regulates RMS cell growth, survival, mTOR signaling and invasion. The pro-oncogenic PAX3-FOXO1 and G9a genes are also regulated by NR4A1 and, these pathways and genes are all inhibited by kaempferol and quercetin. Moreover, at a dose of 50 mg/kg/d kaempferol and quercetin inhibited tumor growth in an athymic nude mouse xenograft model bearing Rh30 cells.

**Conclusion:**

These results demonstrate the clinical potential for repurposing kaempferol and quercetin for clinical applications as precision medicine for treating RMS patients that express NR4A1 in order to increase the efficacy and decrease dosages of currently used cytotoxic drugs.

**Supplementary Information:**

The online version contains supplementary material available at 10.1186/s13046-021-02199-9.

## Background

Flavonoids are phytochemicals produced in fruits, nuts and vegetables that have been directly linked to the health promoting effects of diets enriched in flavonoid compounds. Consumption of total and individual flavonoids have been associated with increased lifetimes and protection from multiple adverse health effects including cardiovascular disease, diabetes and metabolic diseases, neurodegeneration, inflammatory diseases and cancer [1–9]. For example, high dietary intakes of anthocyanins, flavonoids and flavonoid polymers by participants in the prospective Framingham Offspring cohort were correlated with lower risks of dementias including Alzheimer’s disease [10, 11]. Flavonoids exhibit multiple activities and the mechanisms of chemoprevention associated with high dietary intakes of flavonoids are difficult to establish. However, most dietary flavonoids exhibit antioxidant activities and they also enhance the immune system. These effects coupled with other individual flavonoid-dependent responses contribute to their chemoprevention of diseases [12–16].

There is also evidence that diets enriched in flavonoids also protect against development of cancer [2, 4, 16–19] and this is complemented by an extensive literature on the chemotherapeutic effects of individual flavonoids. In vitro and in vivo studies demonstrate that flavonoids inhibit cancer cell growth and migration, and modulate multiple pathways and genes associated with tumorigenesis. The studies on the chemotherapeutic mechanisms associated with flavonoids as anticancer agents primarily have focused on specific functions or genes that are affected. For example, a recent report showed that the flavonoid cardamonin inhibited dextran sodium sulfate (DSS) – induced inflammation in the gut and this anti-inflammatory response was linked to the aryl hydrocarbon receptor (AhR) activity of this compound where cardamonin-duced inhibition of inflammation was dependent on activation of this receptor [20]. Studies in this laboratory have been investigating the pro-oncogenic roles of the nuclear orphan receptor 4A1 (NR4A1, Nur77) in rhabdomyosarcoma (RMS) and other cancer cell lines. In parallel studies, the anticancer activities of bis-indole derived (CDIMs) compounds which are NR4A1 ligands thave been characterized as antagonists [21–27]. NR4A1 has been characterized as a pro-oncogenic factor in many solid tumors and regulates cancer cell growth, survival, migration, invasion, and associated genes [27]. For example, the fusion oncogene PAX3-FOXO1 and G9a have been characterized as highly pro-oncogenic factors in RMS [28, 29] and NR4A1 regulates expression of both genes. In addition, β1-integrin is also an NR4A1-regulated gene and treatment of RMS cells with CDIM/NR4A1 antagonists or knockdown of NR4A1decreased expression of these genes [21, 25]. A recent study reported that the flavonoid kaempferol decreased G9a expression in gastric cancer cells [30] and this was accompanied by growth inhibition, induction of markers of apoptosis and inhibition of mTOR signaling by induced phosphorylation of AMPK. This pattern of responses observed for kaempferol in gastric cancer cells has previously been observed for CDIM/NR4A1 antagonists or NR4A1 silencing in RMS and other cancer cell lines [21–27] and therefore, we hypothesized that kaempferol is an NR4A1 ligand. We have also included quercetin in our studies because it is structurally related to kaempferol and its extensive use as a nutraceutical would facilitate repurposing of quercetin for cancer chemotherapy. This study shows for the first time that both kaempferol and quercetin bind the ligand binding domain (LBD) of NR4A1 and act as NR4A1 antagonists in RMS cells. Both flavonoids inhibit expression of G9a, PAX3-FOXO1, and other pro-oncogenic NR4A1-regulated genes/pathways. Kaempferol and quercetin also inhibited tumor growth in an athymic nude mouse model in vivo suggesting that these nutraceuticals can be repurposed and used in a precision medicine/nutrition approach for treating RMS patients and possibly patients with other cancers that express NR4A1.

## Materials and methods

### Cell lines, reagents and antibodies

The Rh30 cell line was purchased from American Type Culture Collection (Manassas, VA) and was maintained in RPMI medium. The Rh41 cell line was a generous gift from Mr. Jonas Nance, Texas Tech University Health Sciences Center- Children’s Oncology Group (Lubbock, TX) and was maintained in IMDM medium. Both RPMI and IMDM media were supplemented with 10% fetal bovine serum (FBS). Cells were maintained at 37^ο^C temperature in presence of 5% CO_2._ The summary of the reagents/antibodies and oligo sequences used are listed in Supplemental Tables 1 and 2 respectively. Both kaempferol and quercetin were dissolved in 100% DMSO. Rh30 and Rh41 cell lines were treated with the desired concentrations of flavonoids. Knockdown studies by RNA interference (siNR4A1) were carried out essentially as described [21, 22]. The control (DMSO) experiments are indicated and for quantification, DMSO values were set at 1.0 or 100% and treatment-related response are compared to the DMSO value. DMSO was used throughout as the reference control.

### Direct binding assay

The quenching of NR4A1 tryptophan fluorescence by direct ligand binding was carried out essentially as described [31]; the ligand binding domain (LBD) of NR4A1 (0.5 μM) in buffer was incubated with different concentrations of ligands and the fluorescence was obtained using an excitation wavelength of 285 nm (excitation slit width = 5 nm) and an emission wavelength range of 300-420 nm (emission slit width = 5 nm). Ligand binding K_D_ values (not IC_50_ values) were determined by measuring concentration-dependent NR4A1 tryptophan fluorescence intensity at emission wavelength of 330 nm [31].

### Bis-ANS displacement assay

Bis-ANS (Molecular Probes, Inc./ThermoFisher) is essentially non-fluorescent in aqueous solution, however, bisANS fluorescence increases significantly upon binding to protein such as NR4A1. The binding affinity (K_D_) and binding stoichiometry (B_max_) of NR4A1/bisANS was determined essentially as described [32]. Ligand binding affinity (K_i_) to NR4A1 was determined by measuring NR4A1/bisANS fluorescence intensity at emission wavelength of 500 nm as described [32]. Ligand/bisANS fluorescence intensities at each ligand concentration was used to correct the NR4A1/bisANS/ligand fluorescence intensity.

### Luciferase assay

Cells (8 X 10^4^) were seeded in a medium supplemented with 10% FBS and were allowed to attach to 12-well plates. After 24 h, Lipofectamine-2000 reagent (50 μmol/L) in reduced serum medium was used to co-transfect those cells with sequence a) 400 ng (UAS)_x5_-Luc and 40 ng Gal4-NR4A1 or b) 200 ng NBRE_x3_-Luc and 20 ng Flag-NR4A1. The Gal4-NR4A1 chimera contains the yeast Gal4 DNA binding domain fused to NR4A1; the (UAS)× 5-luc construct contains 5 tandem Gal4 binding sites and the NBREx3-luc construct contains 3 tandem sites that bind NR4A1 as a monomer. The medium was removed after 6 h and replaced with 2.5% charcoal-stripped FBS supplemented medium containing either DMSO or flavonoids. After 24 h, the cells were lysed and the cell extract was processed for chemiluminescence quantification of luciferase activity. The Lowry protein assay was used to determine the protein concentration in the cell extract which was used to normalize the luciferase activity as described in [33]. Both Gal4-NR4A1 and Flag-NR4A1 that are used for this study contained full length NR4A1 coding sequence. The plasmids used for this study are constructed as described previously [26, 31, 33].

### Cell survival (XTT) assay

Cells (1 X 10^4^) were seeded in 10% FBS containing medium and were allowed to attach to 96-well plates. After 24 h, the medium was replaced with a fresh medium containing 2.5% stripped charcoal serum supplied with either DMSO or flavonoids. The XTT cell viability kit (Cell Signaling Technology, Danvers, MA) was then used and the manufacturer’s protocol was followed to calculate the percentage of cell survival.

### Western blot analysis

Cells treated with DMSO or flavonoids were lysed and the protein concentrations in cell extracts were quantified using the Lowry protein assay. After normalization, an equal amount of protein was loaded and allowed to run on an SDS polyacrylamide gel. The proteins from the gel were transferred to a PVDF membrane, blocked, and incubated with the primary antibodies (overnight) followed by secondary antibodies (2 h). The HRP-substrate was then added to the membrane and the expression of the protein of interest was detected using Kodak 4000 MM Pro image station (Molecular Bioimaging, Bend, OR).

### Migration (scratch) assay

Cells (3 X 10^5^) were seeded and were allowed to attach. After 24 h, the medium was removed and a scratch was made on the surface using a sterile 200 μl pipette tip. The dead cells were then removed by washing the cells with PBS (2x). The medium supplemented with 2.5% charcoal stripped FBS that contained either DMSO or the desired concentration of flavonoids were then added to the cells. After 24-48 h, the medium was removed, replaced with PBS and the pictures of migrated cells were taken using an Evos digital inverted microscope.

### Boyden chamber invasion assay

Cells (2 X 10^5^) were seeded and were allowed to attach to the cell culture inserts inside wells of cell culture plates. After 24 h, the medium was removed and replaced with the fresh medium supplemented with 2.5% charcoal stripped FBS that contained either DMSO or the desired concentration of flavonoids. After 48 h, cells were trypsinized, counted and 75,000 cells that were suspended in 2.5% FBS supplemental medium were allowed to invade through the matrigel matrix in the Boyden chamber towards the medium containing 10% FBS. After 24 h, the invaded cells trapped on the lower surface of the cell culture inserts were fixed, stained and counted. At least 3 replicates were performed for each treatment group.

### Spheroid invasion assay

Rh41 cells (3 X 10^3^) were seeded in 200 μl 10% FBS supplemented medium in a low attachment round bottom 96 well plate. After 24 h, when the spheroid had formed, 100 μl of medium was gently removed and the plate was allowed to chill on ice. A 100 μl of matrigel was then added to each well without disturbing the spheroid while the plate was still on the ice. The cells were then incubated at 37^ο^C for an hour. A 100 μl of flavonoids (3X the desired final concentration) was then gently added to each well. The cells were then incubated at 37^ο^C for 24 to 48 h. After flagella-like invading structures have developed from the spheroids, the pictures were then taken using an Evos digital inverted microscope. If the flagella-like invading structures are transparent and are difficult to capture in a picture, MTT (3-[4,5-dimethylthiazol-2-yl]-2,5 diphenyl tetrazolium bromide) can be used for staining purposes. If the spheroids need to be kept for a longer period, formaldehyde can be used for fixation.

### PCR

Cells (3 X 10^5^) were seeded in a 10% FBS containing medium and were allowed to attach to 6-well plates. After 24 h, the medium was removed and replaced with 2.5% charcoal stripped FBS supplemented medium that contained either DMSO or flavonoids. The manufacturer’s protocol for the Zymo Research Quick-RNA Miniprep kit (Irvine, CA) was then followed to lyse the cells and extract RNA. The RNA concentration in the extract was then determined, normalized and the high capacity cDNA reverse transcription kit (Thermo Fisher Scientific, Waltham, MA) was used to prepare cDNA from the isolated RNA. The amfiSure qGreen Q-PCR master mix (genDEPOT, Katy, TX) was then used to quantify the expression of mRNA of the gene of interest by quantitative real-time PCR. The human TATA binding protein mRNA was used as a control.

### Overexpression/ rescue experiments

Cells (3 X 10^5^) were seeded on six-well plate in a medium supplemented with 10% FBS and were allowed to attach overnight. They were then transfected with 200 ng Flag-NR4A1 (overexpression/OE) or with the empty vector (EV) using Lipofectamine-2000 reagent (50 μmol/L) in reduced serum medium. After 24 h, these cells were treated with 25 μM kaempferol or quercetin. Twenty-four hours later, the cells were lysed, RNA was extracted and RT-PCR was performed and the total mRNA of desired genes were quantified relative to human TATA binding protein mRNA as outlined in “PCR” in Materials and methods.

### Chromatin immunoprecipitation assay

The manufacturer’s protocol for the ChIP-IT express enzymatic kit (Active Motif, Carlsbad, CA) was followed to perform this assay. Rh30 cells were seeded and allowed to attach for 24 h, then treated with DMSO or flavonoids for 24 h and fixed using formaldehyde. The cross-linking reaction was stopped with glycine and the cells were lysed and nuclei were collected, sonicated, and sheared to collect chromatin fragments. These chromatin fragments were immunoprecipitated with protein specific antibodies in presence of protein G-conjugated magnetic beads. The chromatin fragments were then eluted, the protein-DNA crosslinks were reversed and digestion with protein K was performed to obtain ChIP DNA. The primers designed for specific genes (Supplemental Table 2) were then used to perform PCR with the ChIP DNA and the amplified promoter fraction was resolved on 2% agarose gel in presence of ethidium bromide (Denville Scientific, Metuchen, NJ).

### Immunohistochemistry (IHC)

Tumor tissues were fixed in formaldehyde, embedded in paraffin, sectioned at 4 μM and then mounted on charged slides. These slides were deparaffinized in xylene and rehydrated through graded alcohols. Antigen retrieval was then performed and the slides were washed with Tris buffer. The IHC procedure was then performed on an automated platform (intelliPATH FLX, Biocare Medical, Pacheco, CA). All incubations were carried out at room temperature. Endogenous peroxidase activity was blocked by incubating the slides with 3% hydrogen peroxide for 10 min. A non-serum blocking reagent (Background Punisher, Biocare Medical) was then used to block non-specific protein binding. The Ki-67 antibody (Biocare Medical) was diluted 1:200 and incubated for 50 min and then a polymer detection reagent (Mach 2 HRP Polymer, Biocare Medical) was applied for 25 min. The sites of antigen-antibody interaction were visualized by incubating slides with a DAB chromogen (ImmPACT DAB substrate kit, peroxidase, Vector Laboratories, Burlingame, CA) for 5 min. Mayer’s hematoxylin was used to counterstain the sections. The slides were then dehydrated in 100% alcohol and cleared with xylene. The sections were coverslipped with a permanent mounting medium (Permount Mounting Medium, Electron Microscopy Sciences, Hatfield, PA). IHC images for Ki-67 staining were captured on a Zeiss Axio Imager.M2 motorized microscope using a 20x/0.8 NA PlanApo objective lens (Carl Zeiss Microscopy, LLC, Thornwood, NY).

#### Live cell imaging

For imaging of live RMS cells following treatment, cells were grown on 2-well Nunc™ Lab-Tek™ II Chambered Coverglass slides with a No. 1.5 borosilicate coverglass and imaged using a motorized Zeiss Axiovert 200 MOT with a 20X 0.8 NA objective lens and DIC optics, a Roper Scientific Photometrics CoolSnap HQ Microscope Camera and incubator providing temperature and CO_2_ control.

### Animal studies

All the protocols for the animal studies were approved by the Institutional Animal Care and Use Committee (IACUC) at Texas A&M University. Three to 4 week old female athymic nude mice were purchased from Charles River Laboratories (Wilmington, MA) and were housed in the Laboratory Animal Resources and Research facility, Texas A&M University. Mice were allowed to acclimatize for a week and fed the standard chow diet. Two million Rh30 cells suspended in 100 μl of 1:1 matrigel and PBS solution were injected in each flank of the mouse subcutaneously. When the tumor size was palpable (~ 50 to 100 mm^3^ in size), the mice were randomly divided into control and treatment groups. Each mouse in the control group was administered 100 μl of DMSO: corn oil (1:4) solution by intraperitoneal injection every day. Each mouse in the treatment group was injected with 100 μl of 50 mg/kg flavonoid prepared in DMSO: corn oil (1:4) solution by intraperitoneal injection every day. The mice were weighed and a Vernier Caliper was used to calculate their tumor volume (V = L*W*W/2 mm^3^) every week. After the third week of drug administration, mice were sacrificed and tumors were removed and weighed. A small piece of tumor was homogenized in the lysis buffer and its extract was used for western blot analysis.

### Statistical analysis

The statistical significance of differences between the treatment groups was determined by Student’s t-test. Each assay was performed in triplicate and the results were presented as means with error bars representing 95% confidence intervals. Data with a *P* value of less than 0.05 were considered statistically significant.

## Results

### NR4A1 binding and transactivation induced by flavonoids

**Fig. 1:**
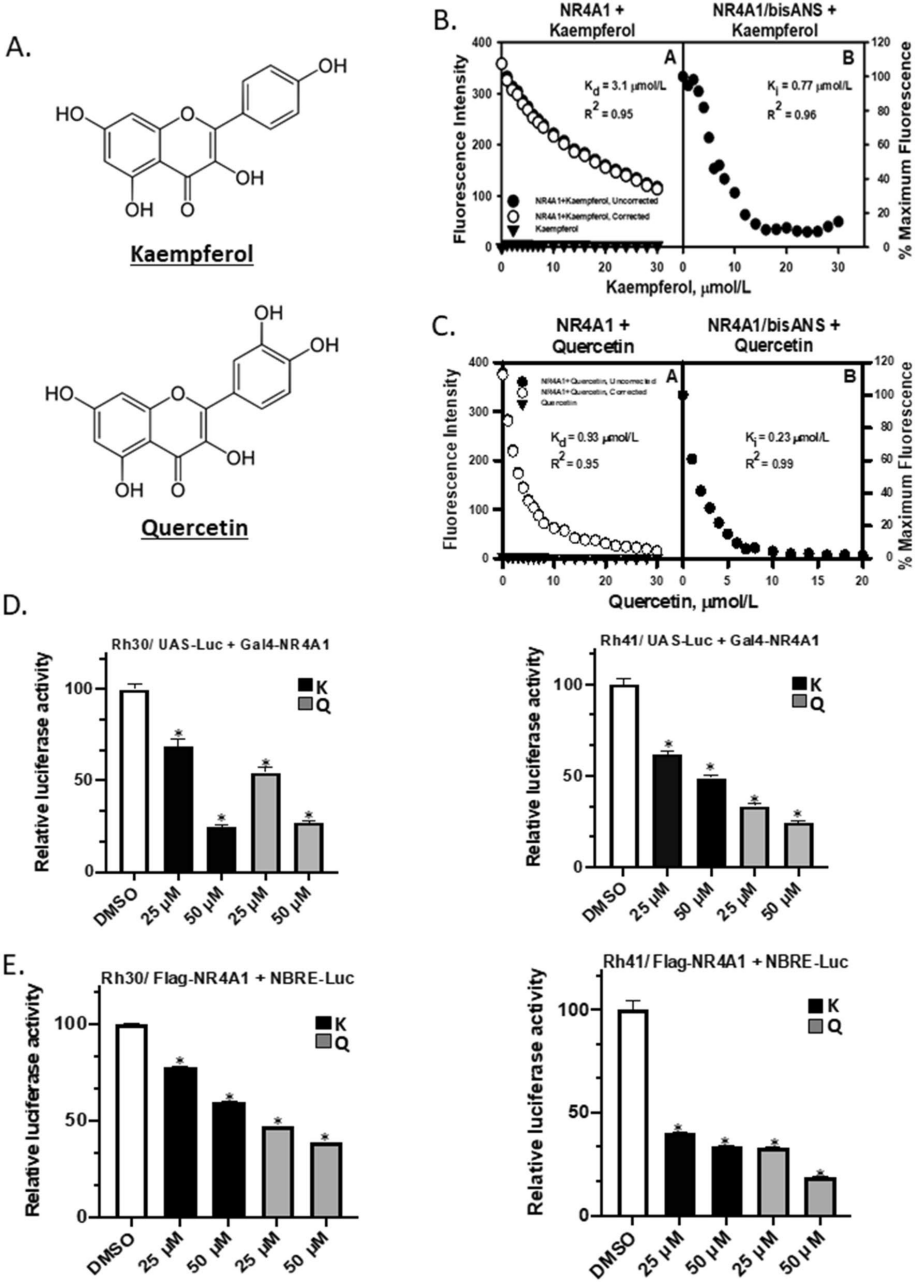
Kaempferol and quercetin bind NR4A1 and inhibit NR4A1-dependent transactivation. **A** Structures of kaempferol and quercetin. Different concentrations of kaempferol (**B**) and quercetin (**C**) were incubated with the ligand binding domain (LBD) of NR4A1 and binding was determined in fluorescent quenching direct binding or a competitive displacement (of bis-ANS) assay as outlined in the Materials and methods. Rh30 and Rh41 cells were transfected with **D** UAS-luc/Gal4-NR4A1 or **E** an NBRE-luc/flag-NR4A1 constructs and after treatment with kaempferol (K) or quercetin (Q) for 24 h, luciferase activity was determined and normalized to the DMSO values as outlined in the Materials and methods. Results are expressed as means ± SD for at least 3 replicated determinations and significant (*p* < 0.05) inhibition is indicated (*)

The histone methyltransferase (EHMT2) G9a is an NR4A1-regulated gene in RMS [21] and the observation that kaempferol decreased expression of G9a in gastric cancer cells [28] suggested that kaempferol may be an NR4A1 ligand acting as an antagonist. In this study we used kaempferol and the flavonoid quercetin (Fig. 1A) and investigated their direct binding with NR4A1 in vitro. Incubation of kaempferol and structurally related quercetin with the ligand binding domain (LBD) of NR4A1 resulted a concentration-dependent quenching of the fluorescence of Trp in the LBD of NR4A1 with K_D_ values of 3.1 and 0.93 μM respectively (Fig. 1B and C) [33]. Kaempferol and quercetin also displaced the fluorescent probe bis-ANS in a competitive binding assay [34] with Ki values of 0.77 and 0.23 μM respectively (Fig. 1B and C). The effects of kaempferol and quercetin on NR4A1-dependent transactivation were also investigated by transfecting cells with a yeast Gal4-NR4A1 construct containing the Gal4 DNA binding domain and NR4A1 and a Gal4-responisve construct containing 5 tandem yeast Gal4 responsive elements linked to a luciferase reporter gene (UASx5-luc). Kaempferol and quercetin also decreased transactivation in Rh30 and Rh41 cells transfected with the Gal4-NR4A1 chimera and a reporter gene (UASx5-luc) construct (Fig. 1D). In addition, kaempferol and quercetin decreased transactivation in Rh30 and Rh41 cells transfected with an NBRE-luc reporter plasmid containing 3 tandem NBRE sites (Fig. 1E). The NBRE-luc construct is NR4A1-responsive and binds the NR4A1 monomer. Thus, like the CDIM/NR4A1 antagonists both kaempferol and quercetin directly bound NR4A1 and antagonized NR4A1-dependent transactivation in Rh30 and Rh41 cells.

### Inhibition of RMS cell growth, survival, migration and invasion by flavonoids

**Fig. 2:**
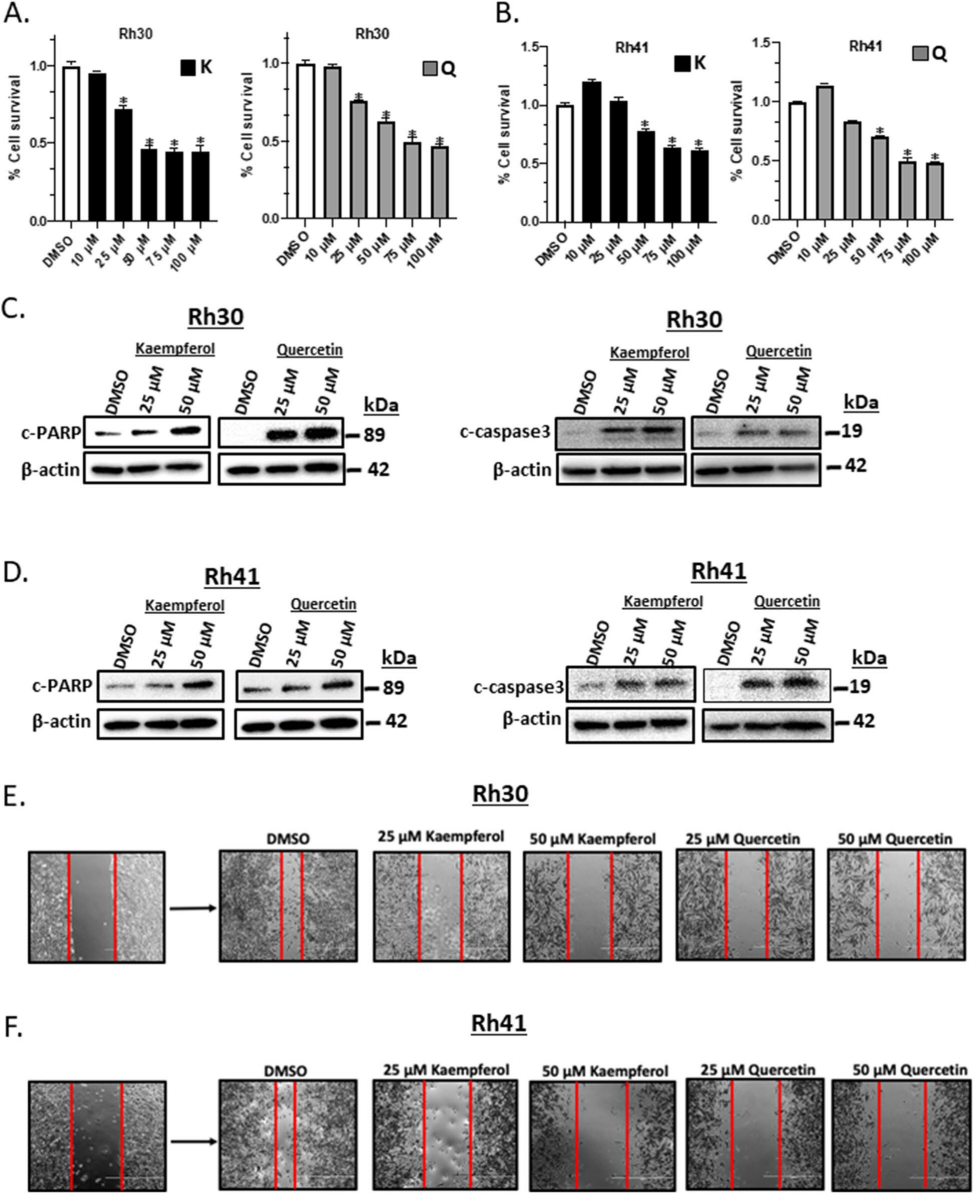
Kaempferol and quercetin inhibit growth, survival and migration of RMS cells. Rh30 (**A**) and Rh41 (**B**) cells were treated with different concentrations of kaempferol (K) and quercetin (Q) for 24 h and cell survival was determined and normalized to the DMSO values as outlined in the Materials and methods. Rh30 (**C**) and Rh41 (**D**) cells were treated with kaempferol and quercetin for 24 h and whole cell lysates were analyzed by western blot analysis as outlined in the Materials and methods. Rh30 (**E**) and Rh41 (**F**) cells were treated with DMSO, kaempferol (K) or quercetin (Q) for 24 h and effects on cell migration were determined in a scratch assay as outlined in the Materials and methods. Results (**A**, **B**) are means ± SD for at least 3 determinations and significantly (*p* < 0.05) decreased growth is indicated (*)

**Fig. 3:**
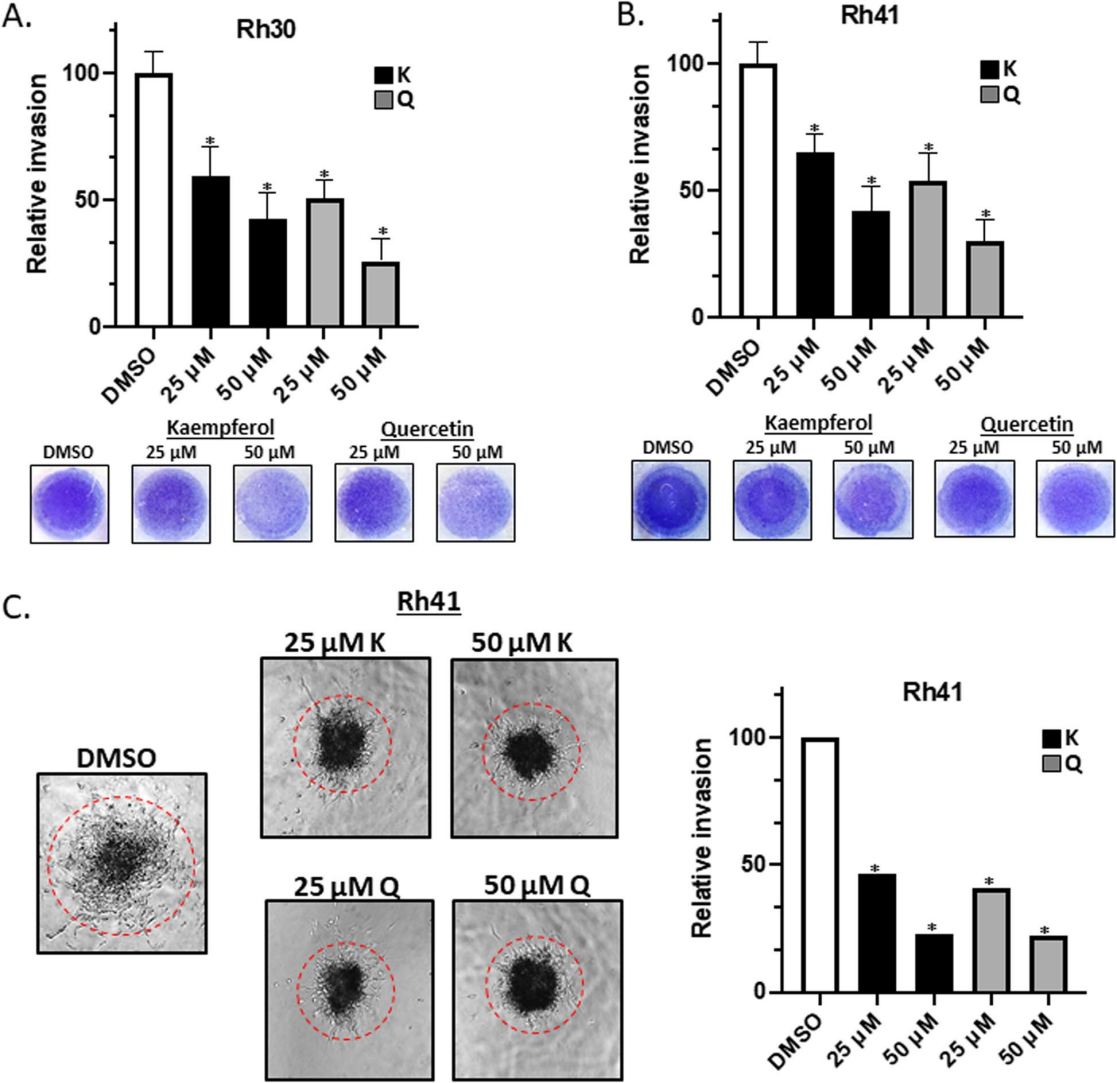
Kaempferol and quercetin inhibit RMS cell migration and invasion. Rh30 Effects of kaempferol and quercetin (24 h treatment) on invasion of Rh30 (**A**) and Rh41 (**B**) cells from medium supplemented with 2.5% fetal bovine serum (FBS) to the medium containing 10% FBS through Matrigel matrix in a Boyden chamber invasion assay were determined and the results were quantified relative to DMSO control. **C** Rh41 cells were grown as spheroids and effects of kaempferol and quercetin on spheroid cell invasion in matrigel matrix were determined as outlined in the Materials and methods. The relative invasion was quantified in comparison to DMSO treated conditions. Results are expressed as means ± SD for at least 3 determinations and significant (*p* < 0.05) inhibition is indicated (*)

Previous studies show that NR4A1 regulates RMS cell growth, survival and invasion, and related genes including the PAX3-FOXO1 fusion oncogene and G9a [21, 25]. Therefore, we further investigated kaempferol and quercetin as antagonists of these NR4A1-dependent pathways/genes. Treatment of Rh30 cells with 10-100 μM kaempferol and quercetin decreased growth (Fig. 2A) and similar effects were observed in Rh41 ARMS cells (Fig. 2B). Treatment of Rh30 cells with 25 or 50 μM kaempferol and quercetin for 24 h significantly induced markers of apoptosis including cleavage of PARP and caspase 3 in Rh30 (Fig. 2C) and Rh41 (Fig. 2D) cells. NR4A1 knockdown or treatment with NR4A1 antagonists also inhibits RMS cell migration [21, 25] and treatment with kaempferol and quercetin for 24 h inhibited migration of Rh30 (Fig. 2E) and Rh41 (Fig. 2F) cells in a scratch assay (quantification in Supplemental Fig. 1). Both flavonoid compounds also inhibited invasion of Rh30 (Fig. 3A) and Rh41 (Fig. 3B) cells in a Boyden Chamber assay and using Rh41 cells as a model 25 and 50 μM kaempferol and quercetin inhibited invasion in a 3-D spheroid invasion model (Fig. 3C). Rh30 cells did not form 3D spheroids in this assay. Thus, like CDIM/NR4A1 antagonists, kaempferol and quercetin inhibited RMS cell growth, survival, migration and invasion.

### Inhibition of NR4A1-regulated genes by flavonoids

**Fig. 4:**
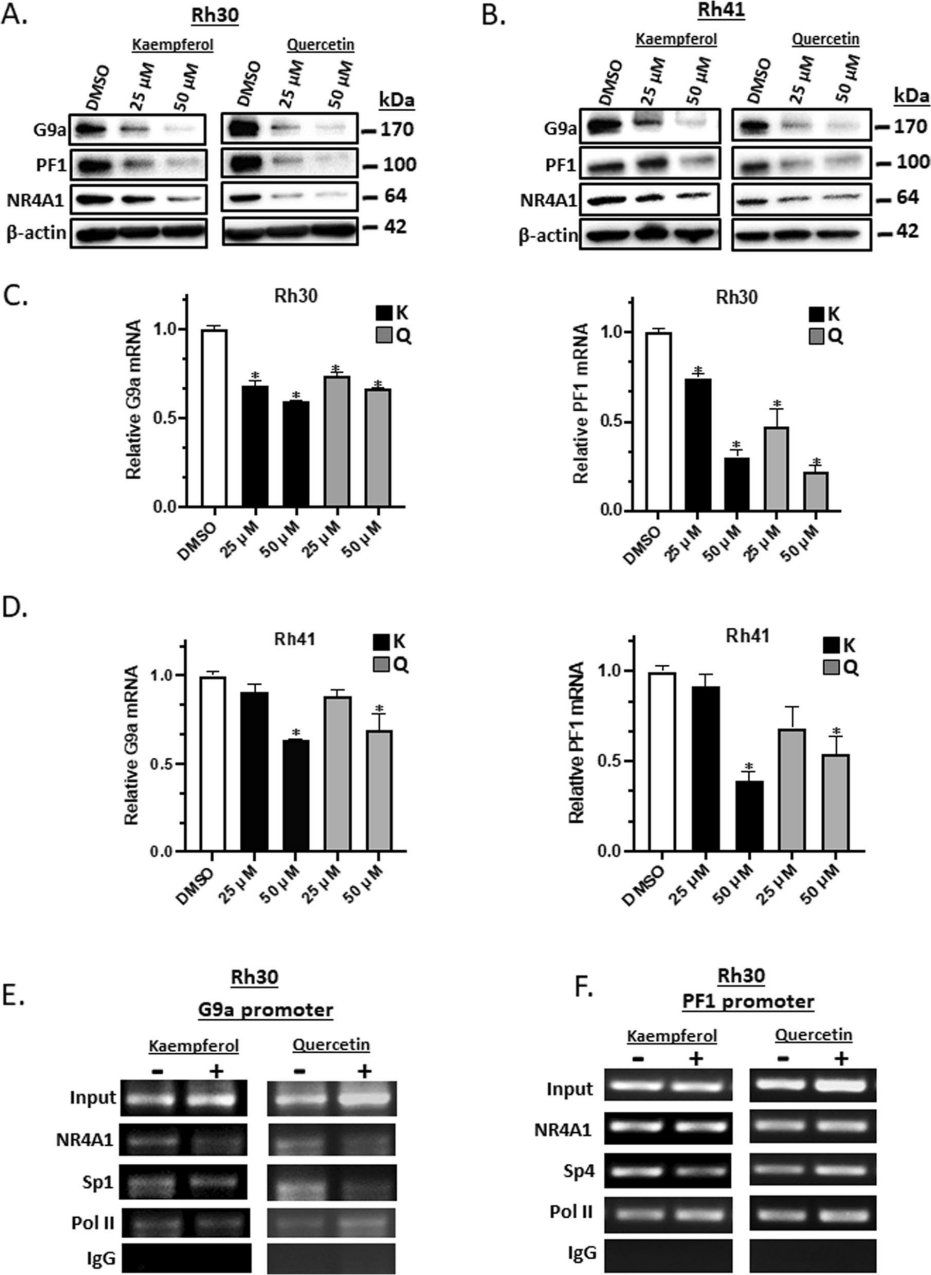
Kaempferol and quercetin downregulate G9a and PAX3-FOXO1 in RMS cells. Rh30 (**A**) and Rh41 (**B**) cells were treated with kaempferol or quercetin for 24 h and whole cell lysates were analyzed by western blots as outlined in the Materials and methods. Rh30 (**C**) and Rh41 (**D**) cells were treated with kaempferol or quercetin for 24 h and G9a and PAX3-FOXO1 mRNA levels were determined by real time PCR as outlined in the Materials and methods. **E** Rh30 cells were treated with 50 μM kaempferol and quercetin for 24 h and analyzed in a ChIP assay and the PAX3-FOXO1 gene (**F**) was also normalized to IgG using the appropriate primers and RT-qPCR as outlined in the Materials and methods. These results are quantified in Supplemental Fig. 3. Results (**C** and **D**) are means ± SD for at least 3 determinations and significant (*p* < 0.05) inhibition is indicated (*)

The histone methyltransferase G9a (EHMT2) and the PAX3-FOXO1 fusion oncogene are regulated by NR4A1 in ARMS cells [21, 25] and treatment of Rh30 cells with 25 and 50 μM kaempferol and quercetin decreased expression of G9a and PAX3-FOX01 gene products (Fig. 4A). Similar results were observed in Rh41 cells (Fig. 4B). We also observed that the flavonoids decreased the levels of NR4A1 proteins and this has previously been observed for some but not all NR4A1 ligands [21, 25] and the rationale for these ligand-dependent effects is not understood and is also observed for ligands that bind other nuclear receptors. Kaempferol and quercetin also decreased expression of G9a and PAX3-FOXO1 mRNA levels in Rh30 (Fig. 4C) and Rh41 (Fig. 4D) cells demonstrating that both flavonoids antagonized NR4A1-dependent gene expression in RMS cells. We also investigated rescue experiments in which quercetin- and kaempferol-dependent decrease in PAX3-FOXO1 and G9a mRNA in Rh30 and Rh41 cells was reversed by overexpression (OE) of NR4A1 compared to effects of an empty vector (EV) control. Overexpression of NR4A1 partially reversed the effects of kaempferol and quercetin on G9a and PAX3-FOXO1 gene expression and most of the effects were significant (Supplemental Fig. 2). Both the G9a and PAX3-FOXO1 promoters contain GC-rich Sp binding sites and are regulated by NR4A1/Sp where NR4A1 acts as a cofactor [21, 25]. Results of ChIP assays in (Fig. 4E, F and Supplemental Fig. 3) demonstrate association of NR4A1 and Sp1 or Sp4 with the G9a and PAX3-FOXO1 promoters respectively in untreated cells and treatment with either kaempferol or quercetin did not significantly increase or decrease NR4A1 or Sp association with the G9a and PAX3-FOXO1 promoters.

**Fig. 5:**
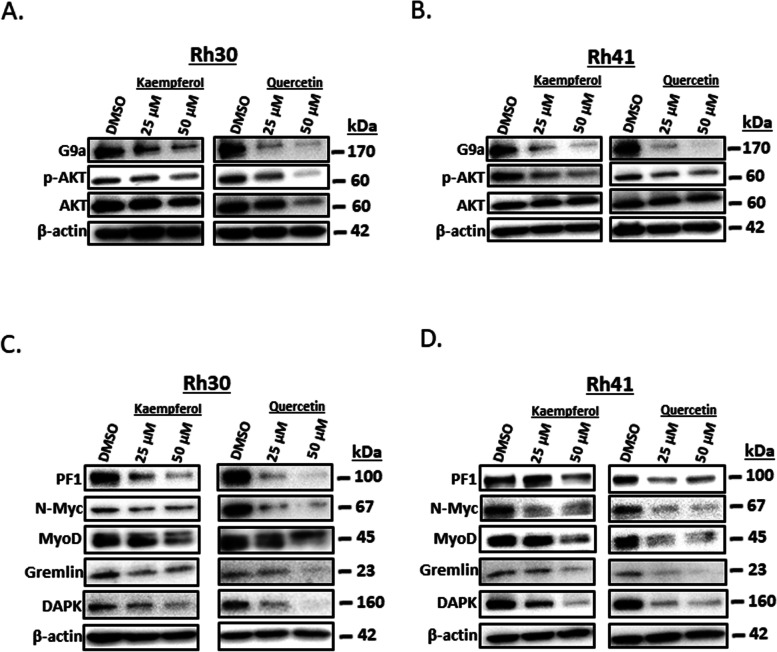
Kaempferol and quercetin inhibit expression of G9a- and PAX3-FOXO1 – regulated gene products. Rh30 (**A**) and Rh41 (**B**) cells were treated with kaempferol or quercetin for 24 h and whole cell lysates were analyzed by western blot analysis as outlined in the Materials and methods. A similar protocol was used to determine expression of PAX3-FOXO1 downstream gene products in Rh30 (**C**) and Rh41 (**D**) cells treated with kaempferol or quercetin for 24 h

**Fig. 6:**
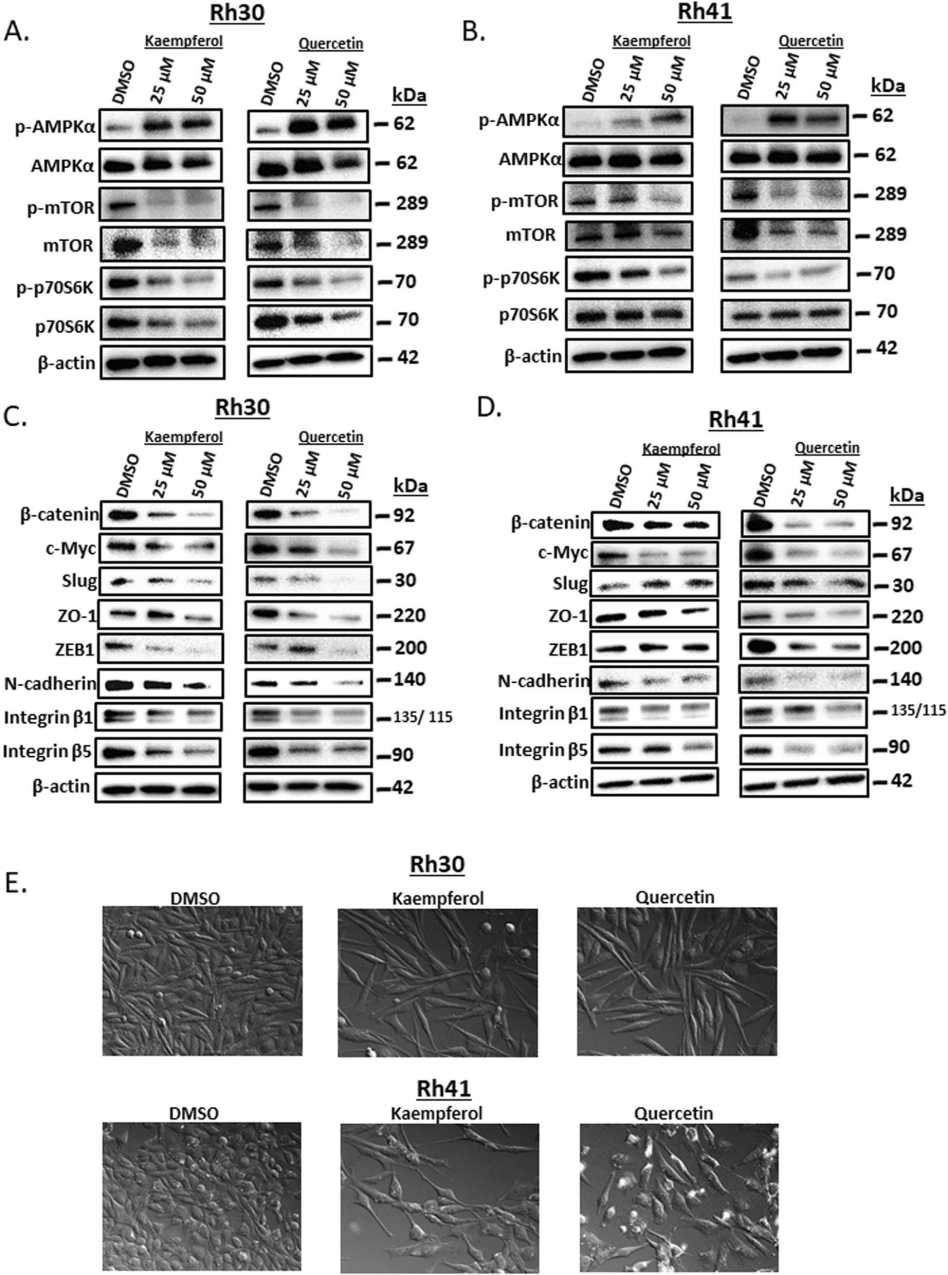
Kaempferol and quercetin act as mTOR inhibitors and induce cell detachment in RMS cells. Rh30 (**A**) and Rh41 (**B**) cells were treated with kaempferol or quercetin for 24 h and whole cell lysates were analyzed by western blots. A comparable protocol was used to determine effects of kaempferol and quercetin on EMT marker gene products in Rh30 (**C**) and Rh41 (**D**) cells. E. Cells were treated with kaempferol and quercetin for 24 h and also transfected with and examined by differential interference contrast imaging as outlined in the Materials and methods

The histone methyltransferase gene regulates Akt phosphorylation in RMS cells [29] and NR4A1 knockdown or treatment with NR4A1 antagonists decreased G9a expression and this resulted in decreased Akt phosphorylation (pAkt). Results illustrated in Fig. 5A and B show that similar effects are observed for kaempferol and quercetin in Rh30 and Rh41 cells respectively. Kaempferol and quercetin also downregulate PAX3-FOXO1 and PAX3-FOXO1 regulated gene products (N-MYc, MyoD, Gremlin and DAPK) in Rh30 (Fig. 5C) and Rh41 (Fig. 5D) cells, and these responses were also previously observed after NR4A1 knockdown or inhibition by CDIM/NR4A1 antagonists [25] demonstrating the activity of both kaempferol and quercetin as NR4A1 antagonists. NR4A1 also regulates mTOR signaling in RMS and other cancer cell [27] lines and NR4A1 knockdown or antagonists inhibit mTOR through reactive oxygen species-dependent activation of AMPK (i.e.: pAMPK) [26, 35–37] and both kaempferol and quercetin induced pAMPK in Rh30 (Fig. 6A) and Rh41 (Fig. 6B) cells and this was accompanied by decreased phosphorylated mTOR and the downstream kinase p70S6K. NR4A1 also regulates gene products associated with attachment and migration [21–25] and treatment of Rh30 (Fig. 6C) or Rh41 (Fig. 6D) cells with kaempferol or quercetin for 24 h inhibits expression of these gene products including β-catenin, c-Myc, Slug, Z0-1, ZEB1, N-cadherin β1 and β5 – integrins. Moreover, image analysis of Rh30 and Rh41 cells after treatment with kaempferol, quercetin for 24 h (Fig. 6E) or after knockdown of NR4A1 (siRNA) (Supplemental Fig. 4) identified in some changes in cell morphology and decreased cell after treatment with the flavonoids or knockdown of NR4A1.

### Kaempferol and quercetin inhibit RMS tumor growth in vivo

**Fig. 7:**
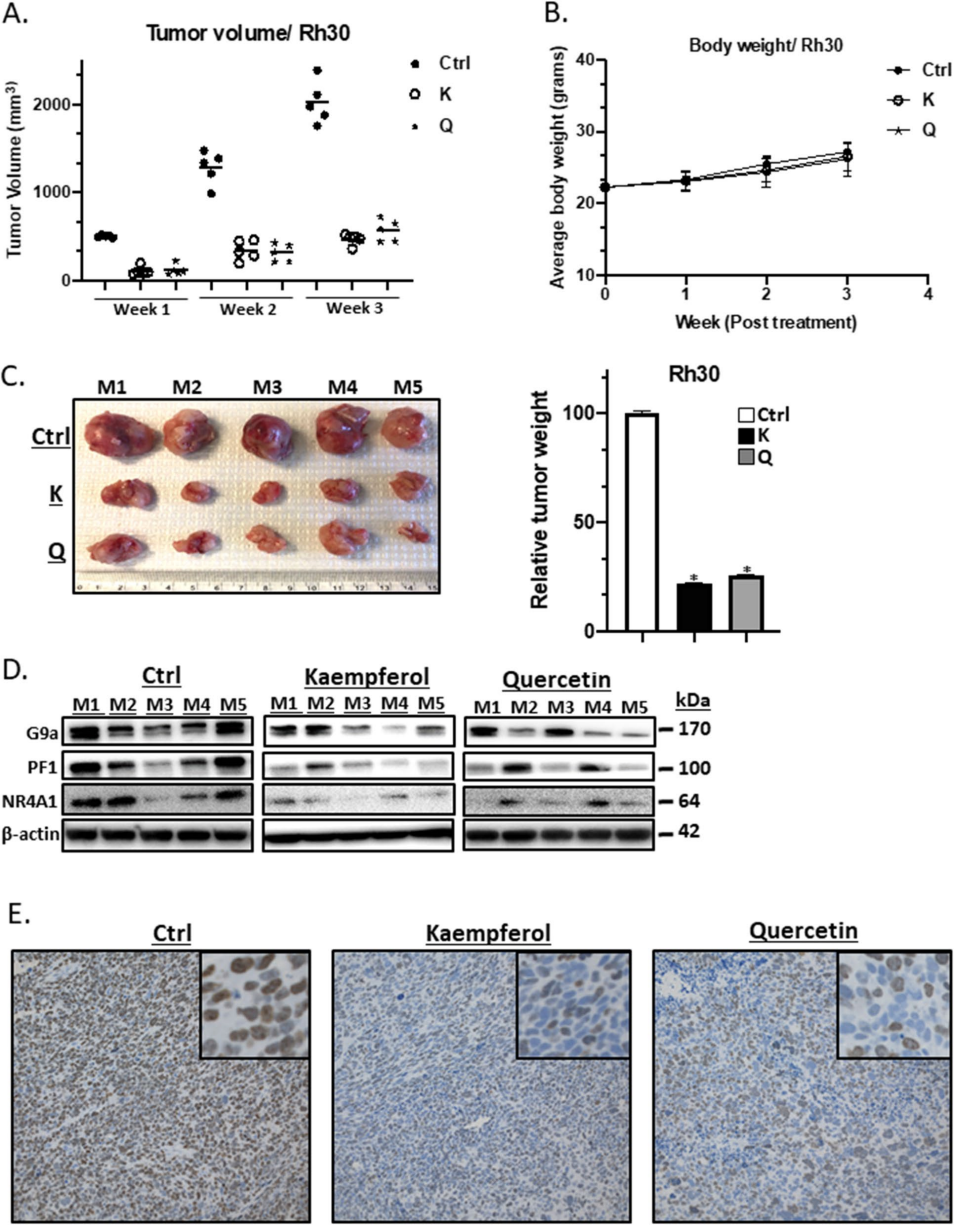
Kaempferol and quercetin inhibit RMS tumor growth. Rh30 cells were injected into flanks of Balb/c athymic nude mice that were treated with kaempferol or quercetin (50 mg/kg/d) by intraperitoneal injection for 3 weeks and effects on **A** cell growth, **B** body weight changes and **C** tumor weights were determined. **D** Tumor lysates were analyzed by western blots for changes in gene expression (relative to the solvent control). **E** Ki67 staining in control and treated tumor sections was determined as outlined in the Materials and methods. Significant (*p* < 0.05) flavonoid-induced effects are indicated

The in vivo anticancer activity of quercetin and kaempferol was investigated in athymic nude mice bearing Rh30 cells as xenografts where cells were injected into the flanking region of mice. At a dose of 50 mg/kg/d, both flavonoids inhibited tumor growth (Fig. 7A) but did not affect body weights (Fig. 7B) over the 3-week treatment period. At sacrifice, tumor weights were decreased (Fig. 7C) and analysis of tumor lysates showed the expression of PAX3-FOXO1 and G9a proteins were decreased (Fig. 7D) and Ki67 staining was also decreased in tumors from mice treated with quercetin and kaempferol (Fig. 7E). The complementary in vitro and in vivo studies indicate that kaempferol and quercetin are NR4A1 antagonists that are highly effective against NR4A1-dependent pro-oncogenic pathways/genes in RMS. These results suggest that NR4A1-active flavonoids can be repurposed from their broad nutriceutical applications for use as targeted agents for clinical treatment of RMS patients with tumors expressing NR4A1.

## Discussion

NR4A1 is a nuclear orphan receptor with no known endogenous ligands and there is increasing evidence that this receptor and other members of this family (NR4A2 and NR4A3) play an important role in maintaining cellular homeostasis and in pathophysiology [27, 38, 39]. NR4A sub-family members are typically induced by cellular stressors and in many diseases, including solid tumors where NR4A1 or other NR4A members are elevated and are potential drug targets. The role of NR4A in cancer is somewhat paradoxical [27]; in many blood-derived tumors NR4A is a tumor suppressor and levels are low. Therefore, agents that induce NR4A1 and its nuclear export are potential therapeutics since the extranuclear receptor can form a proapoptotic NR4A1-bcl2 complex. In contrast, nuclear NR4A1 is pro-oncogenic in solid tumors and regulates cell growth, survival, migration/invasion and related genes [21–27, 31, 35–37]. Studies on the NR4A1 antagonist activities of CDIMs demonstrate that treatment of colon, lung, breast, pancreatic, kidney, RMS, and endometrial cancer cells with CDIM/NR4A1 antagonist inhibited the pro-oncogenic NR4A1-regulated functional responses (rev in 27). Moreover, the effects observed after treatment with CDIMs were comparable to those observed after NR4A1 knockdown.

RMS is a cancer primarily diagnosed in adolescents and accounts for 5% of all pediatric cancers and 50% of soft tissue sarcomas in children with an overall incident rate of 4.5 × 10^6^ [40–42]. Embryonal RMS (ERMS) and alveolar RMS (ARMS) are the two major classes of RMS in children and adolescents and differ with respect to their histology, genetics, treatment, and prognosis [43, 44]. ERMS accounts for over 60% of RMS patients and is associated with the loss of heterozygosity at the 11p15 locus [43]. ERMS patients have a favorable initial prognosis; however, the overall survival of patients with metastatic ERMS is only 40% [44]. ARMS occur in approximately 20% of RMS patients and is associated with translocations from the fusion of *PAX3* or *PAX7* with the Forkhead gene *FOXO1* resulting in formation of pro-oncogenic gene products [45, 46]. ARMS patients have a poor prognosis and patient survival is < 10% for metastatic ARMS [47]. Treatments include radiotherapy, surgery, and chemotherapy with cytotoxic drugs and/or drug combinations; RMS patients that survive current cytotoxic drug therapies have > 95% increased risk for several diseases as adults ≥45 years of age [48]. Thus, there is a critical need for development of new therapeutic regimens for treating childhood RMS and for developing innovative therapies for treating ARMS patients since the current cytotoxic drug therapies have limited effectiveness and cause health problems later in life. Our previous research has identified NR4A1 as a new drug target for treating RMS. NR4A1 is overexpressed in RMS and correlates with expression of PAX3-FOXO1 in ARMS patients and treatment with synthetic CDIMs that are NR4A1 antagonists are highly effective in both cell culture and in vivo studies. The efficacy of NR4A1 antagonists is due, in part to their suppression of NR4A1-regulated mTOR signaling, PAX3-FOXO1, β1-integrin and downstream gene products and the histone methyltransferase G9a [21, 25]. The origins of this study were based on a recent report showing that the flavonoid kaempferol downregulated G9a in gastric cancer cells [30] and we hypothesized that kaempferol and possible other flavonoids may be NR4A1 ligands that act as receptor antagonists.

Results in Fig. 1 confirm that kaempferol and quercetin directly bind NR4A1 and competitively displace a fluorescent bound ligand (bis-ANS) and they also inhibit NR4A1-dependent transactivation. These results coupled with the effects of kaempferol and quercetin on cell growth, survival, migration and invasion (Figs. 1, 2 and 3) are also observed in RMS cells after NR4A1 knockdown or treatment with CDIM/NR4A1 antagonists [21–27].

PAX3-FOXO1 and G9a are genes that play pro-oncogenic roles in RMS [28, 29] and these genes are regulated by NR4A1 which acts as a co-factor to enhance Sp1- or Sp4- mediated gene expression through NR4A1/Sp1/4 binding GC-rich promoter elements [21–25]. This mechanism of NR4A1/Sp gene regulation is not uncommon and is observed for many other nuclear receptors [49]. Both kaempferol and quercetin decrease expression of PAX3-FOXO1 and G9a mRNA and proteins and downstream gene products (Fig. 4). Similar results were observed for activation of pAMPK and inhibition of mTOR signaling and for inhibition of genes associated with cell attachment/migration and accompanying morphological changes (Figs. 5 and 6). We also observed that at doses of 50 mg/kg/d quercetin and kaempferol were potent inhibitors of RMS tumor growth in athymic nude mice bearing Rh30 cells injected into their flanking regions (Fig. 7). The complementary results of cell culture and in vivo studies demonstrate for the first time that kaempferol and quercetin are NR4A1 ligands that act as antagonists in RMS cells and mimic the effects of NR4A1 knockdown by RNA interference [21–25]. These results suggest that NR4A1-active flavonoids can be repurposed for clinical applications in the treatment of RMS and possibly other cancers where NR4A1 is a potential drug target. This type of precision medicine/nutrition approach for using flavonoids would specifically target patients that overexpress NR4A1 and could be used clinically for increasing the efficacy and decreasing the dose of currently used cytotoxic therapies.

## Conclusion

RMS patients are routinely treated with cytotoxic drug combinations which have limited effectiveness and induce serious adverse health conditions later in life. NR4A1 is a pro-oncogenic factor for RMS and synthetic NR4A1 antagonists are highly effective inhibitors of growth and invasion in both cell culture and in vivo mouse models. In this study we have identified for the first-time two flavonoids that are widely used in nutriceuticals as NR4A1 ligands that act as antagonists to block NR4A1-regulated responses in RMS. The results suggest that repurposing quercetin and kaempferol for clinical applications in treating RMS patients would not only enhance the effectiveness but also lower the dosages of currently used cytotoxic agents for treating patients with this deadly pediatric tumor.

## Supplementary Information

**Additional file 1.**

## Data Availability

Not applicable.

## Abbreviations

RMS: Rhabdomyosarcoma
NR4A1: Nuclear receptor 4A1
AMPK: AMP-activated protein kinase
RPMI: Roswell Park Memorial Institute
IMDM: Iscove’s Modified Dulbecco’s Media
FBS: Fetal bovine serum
DMSO: Dimethyl sulfoxide
PBS: Phosphate buffered saline
IACUC: Institutional Animal Care and Use Committee
LBD: Ligand binding domain
EHMT2: Euchromatic Histone Lysine Methyltransferase 2
pAkt: Akt phosphorylation
ERMS: Embryonal Rhabdomyosarcoma
