# Supplementary material for: Flavonoids kaempferol and quercetin are nuclear receptor 4A1 (NR4A1, Nur77) ligands and inhibit rhabdomyosarcoma cell and tumor growth
Source: J Exp Clin Cancer Res. 2021 Dec 14;40:392. doi: 10.1186/s13046-021-02199-9 (PMC8670039; doi:10.1186/s13046-021-02199-9)
Supplement: Supplementary file 1 — Additional file 1. [file 13046_2021_2199_MOESM1_ESM.docx]

|  | **Reagents/Antibodies** | **Purchased From:** |
| --- | --- | --- |
| 1. | FBS, Trypsin, RPMI, IMDM, Bis-ANS molecular probes | Thermofisher Scientific (Waltham, MA) |
| 2. | Kaempferol | MedChemExpress LLC (Monmouth Junction, NJ) |
| 3. | Quercetin | Pfaltz & Bauer Inc. (Waterbury, CT) |
| 4. | C-parp (5625), c-caspase3 (9661), G9a (68851), PAX3-FOXO1 (2880), IgG (2729), p-AKT (4060), AKT (4685), p-AMPKα (50081), AMPKα (5832), p-mTOR (2971), mTOR (2972), p-p70S6K (9205), p70S6K (9202), Slug (9585), ZO-1 (8193), ZEB1 (3396), N-cadherin (13116), β-catenin (8480), Integrin β1, Integrin β5 and HRP-linked secondary antibodies (7074, 7076) | Cell Signaling Technology (Danvers, MA) |
| 5. | NR4A1 antibody | Abcam (Cambridge, UK) |
| 6. | Sp1 (17824), Sp4 (390124), Pol II (47701), N-myc (53993), MyoD (32758), Gremlin (515877), DAPK (136286), c-Myc (sc-40) antibodies | Santa Cruz Biotechnology (Dallas, TX) |
| 7. | β-actin antibody (A5316), oligonucleotides for siRNA interference assay | Sigma-Aldrich (St. Louis, MO) |
| 8. | Lipofectamine-2000, Alexa Flour® 488 annexin V/Dead cell apoptosis kit | Invitrogen (Carlsbad, CA) |
| 9. | Reporter lysis buffer and luciferase reagent | Promega Corporation (Fitchburg, WI) |
| 10. | PCR/ChIP primers | Integrated DNA Technologies (Coralville, IA) |
| 11. | Ethidium bromide | Denville Scientific Inc. (Metuchen, NJ) |
| 12. | Invasion chambers, Matrigel | Corning Inc. (Corning, NY) |
| 13. | Chemiluminescent Immobilon western HRP-substrate | MilliporeSigma (Burlington, MA) |

**Supplemental Table 1:**

**Supplemental Table 2:**

|  | **Oligonucleotides** | **Sequence** |
| --- | --- | --- |
| 1. | Human G9a primer (PCR) | F: 5'-TGGGCCATGCCACAAAGTC-3'  R: 5'-CAGATGGAGGTGATTTTCCCG-3' |
| 2. | Human PAX3-FOXO1 primer (PCR) | F: 5’-CCCACTGCCATGCCGACCTTG-3’  R: 5’-ACGAATTGAATTCTGAGGTGAGAG-3’ |
| 3. | Human NR4A1 primer (PCR) | F: 5’- CACAGCTTGCTTGTCGATGTC -3’  R: 5’- ATGCCGGTCGGTGATGAG -3’ |
| 4. | Human G9a promoter (ChIP) | F: 5’-CAGATGGGGACAGAGACGC -3’  R: 5’-CCCGGAGCATTGCACG-3’ |
| 5. | Human PAX3-FOXO1 promoter (ChIP) | F: 5’-TGCCTGTGCTTCACATTAGC-3’  R: 5’-AATTCCAATAAGAAGGCATCTG-3’ |
| 6. | siNR4A1_C | 5’-CAGUGGCUCUGACUACUAU-3’ |
| 7. | siNR4A1_D | 5’-GAGAGCUAUUCCAUGCCUA-3’ |

**Supplemental Figures**

**Figure 1**.

**K**

**Q**

*****

*****

*****

*****

**K**

**Q**

*****

*****

*****

*****

**Supplemental Figure 1.** Quantitation of the effects of quercetin (Q) and kaempferol (K) on cell migration (A) as illustrated in Figure 2E/2F respectively.

**Figure 2**.


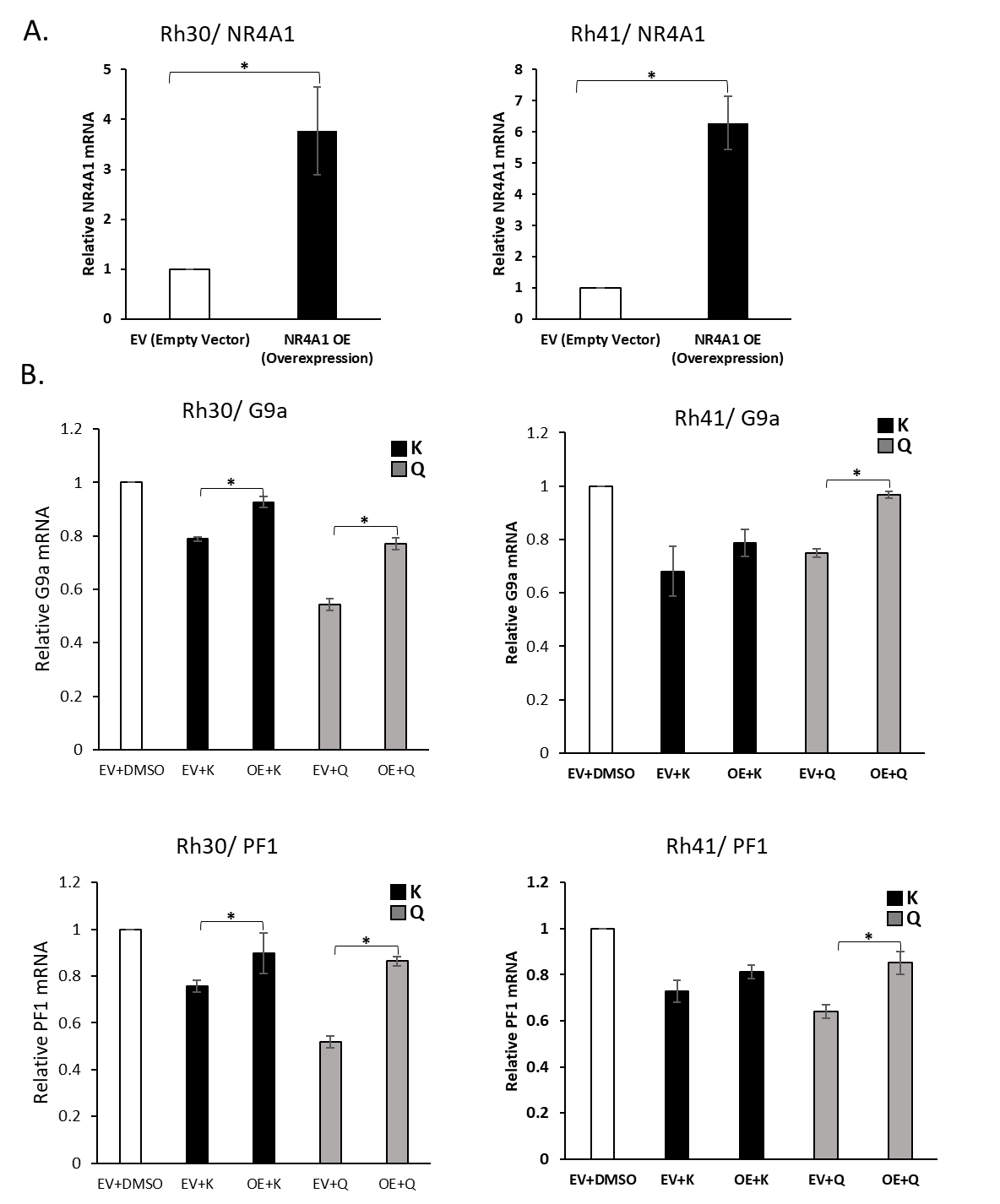


**Supplemental Figure 2. Overexpression and Rescue experiments:** Transfection of (A) Rh30 and Rh41 cells with Flag-NR4A1 (200 ng) showing significant NR4A1 overexpression (OE) in comparison to the cells transfected with empty vector (EV). B. NR4A1 overexpression partially reversed kaempferol and quercetin (25 µM) induced decrease of NR4A1-regulated genes G9a and PAX3-FOXO1 (PF1) in Rh30 and Rh41 cells.

**Figure 3**.


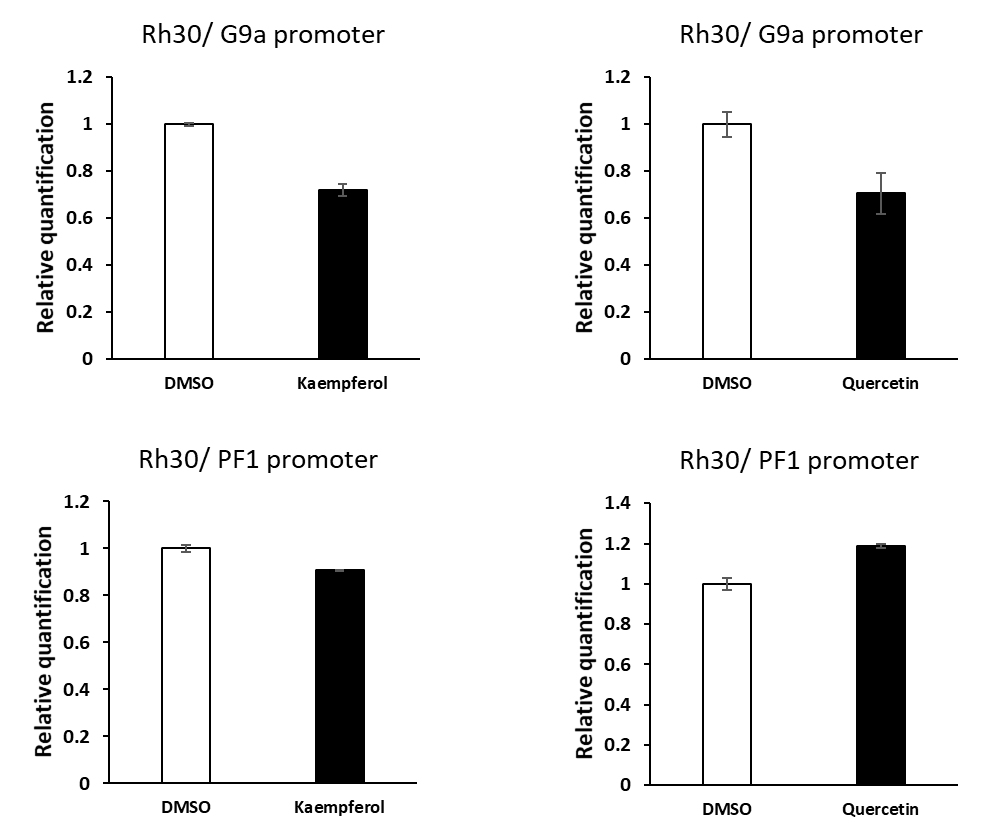


**Supplemental Figure 3.** ChIP quantitation of NR4A1 binding in Rh30 cells: The ChIP assay results (Fig. 4E) showing binding of NR4A1 to the G9a and PAX3-FOXO1 (PF1) gene promoters are quantitated relative to the DMSO control values set at 1.0.

**Figure 4**.


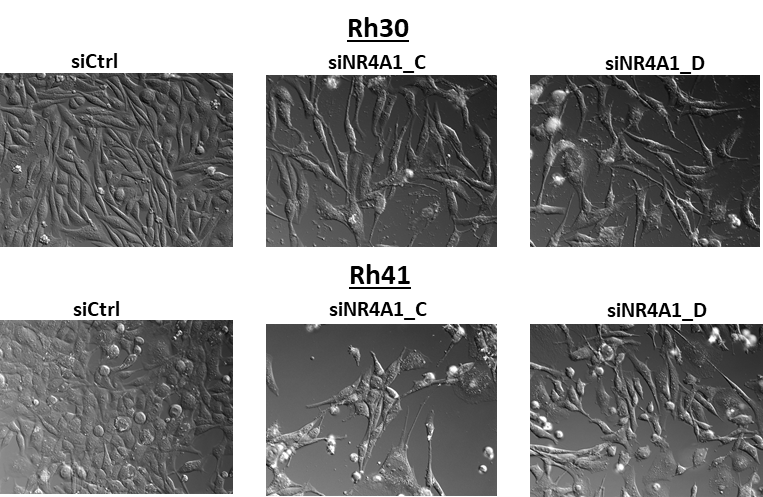


**Supplemental Figure 4.** Cell morphological changes after knockdown of NR4A1 in Rh30 and Rh41 cells.
